# Acute neuroendocrine challenge elicits enhanced cortisol response and parallel transcriptomic changes in patients with migraine

**DOI:** 10.1097/PR9.0000000000001254

**Published:** 2025-05-01

**Authors:** Sahel Kumar, Peter Petschner, Kinga Gecse, Dora Torok, Gabriella Juhasz

**Affiliations:** a Department of Pharmacodynamics, Faculty of Pharmaceutical Sciences, Semmelweis University, Budapest, Hungary; b NAP3.0-SE Neuropsychopharmacology Research Group, Hungarian Brain Research Program, Semmelweis University, Budapest, Hungary

**Keywords:** Migraine, Citalopram, Stress response, Cortisol, RNA sequencing, Gene expression, Neuroendocrine

## Abstract

**Introduction:**

Migraine is a neurological disorder with recurrent attacks characterized by headaches and sensitivity to stimuli. Stress is a significant trigger for attacks; however, molecular mechanisms of the connection are poorly understood.

**Objectives:**

To better characterize such mechanisms, we performed a placebo-controlled, double-blind crossover study with 51 participants (21 patients with migraine without aura and 30 healthy controls).

**Methods:**

Participants received a low-dose citalopram- or placebo challenge on 2 separate days. Prechallenge and postchallenge assessment of cortisol concentrations and transcriptomic changes using RNA-seq was performed from whole blood samples. Analysis of an accidental attack following the citalopram challenge was also conducted.

**Results:**

Neuroendocrine challenge elicited elevated cortisol concentrations at 30 (*P*-value = 0.1355) and 70 minutes (*P*-value = 0.07292) postchallenge in patients with migraine compared with controls. Gene expression analysis showed 10 differentially expressed genes (2 down- and 8 upregulated, *P*-value ≤ 0.005) and 10 dysregulated gene sets (*P*-value ≤ 0.005). Among them, dysregulated *IKBKGP1* and *NKRF* genes and upregulated protein synthesis and translation, carbohydrate metabolism, and, attack-related, glycosylation can be highlighted.

**Conclusion:**

Patients with migraine without aura showed an enhanced cortisol response to a neuroendocrine challenge. This was accompanied by a probable suppression of *NF*κ*B* activity through dysregulation of *NKRF* and an altered immune function. Upregulated carbohydrate metabolism may reflect the elevated cortisol concentrations' stimulating effects on endothelial glycocalyx, playing a role in NO-induced vasodilation, a trigger for migraine attacks. The results suggest the elevated cortisol response may trigger migraine attacks through altered glycocalyx and immune functions.

## Abstract 2

Supplemental Digital Content is Available in the Text.

Patients with migraine without aura exhibit heightened cortisol responses and gene expression changes after a neuroendocrine challenge, suggesting an altered stress response linked to migraine pathophysiology.

## 1. Introduction

Migraine is one of the most disabling disorders in the world. In addition to moderate-to-severe headaches, it is characterized by sensory hypersensitivity and stress sensitivity even between attacks.

Migraine sufferers often cite heightened stress levels as the underlying cause of attacks.^12^ Early studies on small populations demonstrated a temporal connection between stress intensity rating/frequency and migraine attacks^21^ and that 84% of patients with migraine complained about stress as an attack aggravating and precipitating factor.^47^ The latter results were confirmed in a large population of 1750 patients with migraine, where 79.7% of patients reported stress as a trigger for their migraine attacks.^24^ A more recent systematic review by Peroutka, combining 25 different studies confirmed stress as a significant precipitator of migraine in approximately 58% of 7187 subjects.^39^ A recent narrative review found limited evidence for the causality between stressors and migraine attacks but supported associations and suggested that behavioural interventions aiming at reducing stress load may be beneficial for migraine management.^50^ At the same time, a reduction of perceived stress was also associated with migraine attacks in the next 6, 12, and 18 hours, promoting the so-called “let-down” hypothesis, namely, that attacks are elicited by a reduction in stress levels.^30^ A larger and more recent study including 351 patients, however, showed that among reported 2115 valid migraine episodes “let-down” attacks were the smallest proportion.^53^ Taken together, numerous studies pointed to the prominent role of stress as a factor behind migraine attacks either directly, or as a preceding factor with longer latency, leading to the assumption that maladaptive stress responses have a major role in migraine pathophysiology.^6^ Albeit sporadic efforts attempted to bind various stress-related physiological phenomena with migraine attacks,^50^ to our knowledge, no systematic studies tried to identify the exact molecular mechanisms of the connection between stress and migraine attacks in humans.

This may be because environmental stress is recorded in the above studies after the stressor has happened, limiting the analyses to retrospective correlation or descriptive statistics. Neuroendocrine challenges may provide a viable alternative to simulating an endogenous response. For example, an acute citalopram challenge can induce arousal and a neuroendocrine stress response through the release of prolactin, adrenocorticotropic hormone, and cortisol through activation of the hypothalamic-pituitary-adrenal (HPA) axis.^2,5,31,34^ The challenge already provided compelling evidence for altered tryptophan and kynurenine metabolism^18^ and increased neuronal stress sensitivity in migraine without aura (MO); the method is associated with minimal side effects, validating its use as an experimental tool in patients with migraine to test the stress axis.

In the systematic discovery of molecular mechanisms behind various biological phenomena, mRNA sequencing is a valuable tool. It can measure RNA levels relatively simply with unlimited dynamic range, shows high independent replication rates, and provides the direction of change of alterations, and mRNA levels explain a reasonable amount of protein level variation in various tissues.^29,48^ In addition, bioinformatic analysis tools on mRNA data, like gene set enrichment analysis (GSEA),^51^ can deliver estimates of up-/downregulations on a pathway level capturing higher-level alterations in biological processes than individual genes. Therefore, RNA sequencing could deliver complex, omics-based insights into the hypothesized relationship between stress and migraine attacks after a neuroendocrine challenge.

In accordance with the above, our aim in this study was to investigate the transcriptomic alterations in patients with MO compared with healthy controls during an acute stress challenge to elucidate underlying molecular mechanisms of the altered stress response in the interictal period. We measured both cortisol concentrations as indicators of stress response and correlated transcriptomic changes to reveal the molecular associations and potentially testable mechanisms connecting stress and migraine attacks.

## 2. Methods

### 2.1. Participants

This study adhered to the Helsinki Declaration for Research and was approved by the Hungarian ethical committee of the Medical Research Council (number: 23609-1/2011-EKU, December 19, 2011; 23421-1/2015-EKU, May 12, 2015). All participants provided written informed consent before participation. Volunteers were recruited in Hungary through university advertisements, headache clinics, newspaper articles, and online platforms. Episodic MO diagnosis was made by expert neurologists following the International Classification of Headache Disorders third edition. Smoking status, age, sex, history of allergies, pregnancy, breastfeeding, other acute and chronic illnesses, and regular medication use were registered by self-reported questionnaires and during consultation with the neurologist. Zung Self-Rating Depression Scale (SDS) and State-Trait Anxiety Inventory for State and Trait Anxiety scores were also collected. In addition, data regarding monthly headache frequency, years since onset, attack triggers, and average headache severity (MIDAS B^49^) were obtained from the MO population through headache diary and questionnaires. Mental health status was evaluated using the Mini International Neuropsychiatric Interview.^46^

A total of 51 participants were included: 21 diagnosed with MO (5 men and 16 women) and 30 healthy controls (14 men and 16 women). Exclusion criteria included neurological problems (except MO), psychiatric disorders, significant health issues (except the history of allergies in a nonactive state), regular medication use (except contraceptives), pregnancy, and breastfeeding. Furthermore, patients with MO were excluded if they experienced an attack within 48 hours before or 24 hours after sampling. Healthy controls were required to be headache-free within 48 hours before sampling. Participants confirmed abstaining from analgesics 24 hours and caffeine 4 hours before sample collection (with other medications already excluded).

**Figure 1.**
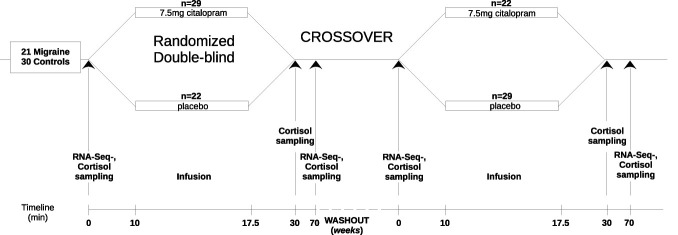
Schematic representation of study design. Over the course of the study, 51 participants underwent a placebo-controlled, randomized, and double-blind crossover design using a placebo- or citalopram challenge. During the challenge days, 3 blood samples for cortisol measurement and 2 for RNA-seq analysis were taken: first, 10 minutes before the challenge (0 minutes); then, they were given a 7.5-mg citalopram infusion or saline at random for 7.5 minutes; participants gave second and third blood samples at 20 and 60 minutes after the challenge started (30 and 70 minutes compared with the first sample). A washout period of varying duration, with a minimum of 12 days occurred before the participants repeated the process once more with the opposite substance given to them.

Both MO and control individuals were assigned to placebo or citalopram challenge randomly in a double-blind fashion. At the first set of measurements, MO and control participants assigned to the citalopram challenge group received a 7.5-minute-long infusion containing a cumulative dose of 7.5 mg citalopram. Those in the placebo challenge group received saline infusion. In the second set of measurements, at least 12 days after the first challenge, people assigned to the citalopram group on the first occasion were assigned to the placebo group and vice versa in a crossover design (Fig. 1). On measurement days, blood was drawn 3 times: prechallenge and postchallenge, at 0, 30, and 70 minutes, with cortisol measurements at all time points, and RNA-sequencing measurements only at 0 and 70 minutes (Fig. 1). The 70-minute postcitalopram sampling time point was selected based on prior studies demonstrating cortisol elevations in healthy individuals.^2,5,31^ On both occasions, participants carried out different psychological tasks including emotional face recognition, monetary incentive delay, and pain anticipation.^17,25,26,52^ The effects of these tasks were not analyzed here, but corrections for sampling day were included as co-variate to exclude potential bias.

### 2.2. Migraine attack in a patient

A patient showed a migraine attack during sampling, with citalopram infusion leading to gradually worsening condition and vomiting. She was administered metoclopramide and metamizol i.v., after sampling. We performed all analysis steps listed below twice: without the inclusion of the patient (Supplementary Table 1, https://doi.org/10.5281/zenodo.14910825) and with the patient (Supplementary Table 1, https://doi.org/10.5281/zenodo.14910825) to assess the impact of the attack.

### 2.3. Cortisol-concentration measurements

The cortisol measurement protocol was previously described in Reference 18. In brief, blood samples were drawn into K3EDTA tubes and centrifuged immediately (10 minutes at 2000 RPM). The resulting plasma was stored at −80°C until analysis. Plasma cortisol concentrations were determined using ELISA kits (NovaTec Immunodiagnostica GmbH [Dietzenbach, Germany]) per the manufacturer's protocol. To align with RNA-sequencing measurements (where the same comparisons were made), we calculated the difference of cortisol-concentration postchallenge minus cortisol-concentration prechallenge for both citalopram (DiffCitalopram) and placebo (DiffPlacebo). We calculated the difference of the differences (DiffOfDiffs = DiffCitalopram − DiffPlacebo) of individuals and compared MO and healthy control DiffOfDiffs values using nonparametric Mann-Whitney *U* test because normality could not be confirmed by visual inspection of data (Supplementary Figure 1, http://links.lww.com/PR9/A289). During analysis, only individuals with valid values for all measurements were considered (19 MO and 29 healthy controls). Significance levels were set at *P*-value < 0.05, and all analyses were conducted using R (v4.0.3).

To investigate baseline and placebo-induced effects, cortisol concentrations between patients and controls prechallenge and postchallenge after placebo were modelled by linear model: lm(Cortisol ∼ Group + Sex + Age + Allergy + Smoking).

### 2.4. Blood sample preparation, RNA-seq, its quality control, and data processing

#### 2.4.1. RNA-seq data handling and analysis

To assess challenge-related transcriptomic changes, blood samples were collected and stored in PAXgene Blood RNA tubes (Qiagen, Venlo, The Netherlands) at −80°C in accordance with the manufacturer's guidelines. Samples of 51 participants underwent purification and extraction using a PAXgene Blood mRNA kit (Qiagen) on a QiaCube instrument, following the manufacturer's instructions. Samples were processed using New England Biolabs (NEB) NEBNext Ultra Directional RNA Library Prep Kit for Illumina, following the protocol specified in the NEBNext Ultra Directional RNA Library Prep Kit for Illumina (NEB #E7420S/L) at GenomeScan (Leiden, The Netherlands). Sequencing was carried out on an Illumina NextSeq 500 instrument (Illumina, San Diego, CA) with 75 bp single-end sequences, following standard operating procedures of Illumina and GenomeScan, as well as ISO standards with a sequencing depth of 20 million reads per sample.

Potentially remaining NEBNext adapters were removed using Trimmomatic (v0.39). Raw data quality control (QC) was performed using FastQC (v0.11.8). All reads exhibited a nucleotide quality score exceeding 30; thus, no samples were excluded based on QC results.

To ensure proper alignment, RNA-seq reads were mapped to the Ensembl human reference GRCh38.96 with HISAT2 (v2.2.1) using default settings and spliced alignment. Transcriptome assembly was performed with StringTie (v2.1.4).

#### 2.4.2. Gene- and pathway-level analysis

For differential gene expression analysis in R (v4.0.3), the Bioconductor package edgeR (v3.30.3) was utilized. Gene set enrichment analysis (GSEA) was done with fgsea (v1.14.0) using C2, C3, and C5 sets from the Molecular Signatures Database (MsigDB) datasets (2023v1). C2 set contains pathway-based gene sets compiled from disparate sources such as online databases, articles from PubMed, and knowledge about different domains. The C3 collection consists of gene sets derived from transcription factor target genes and microRNAs (miRNAs). Furthermore, C5 sets contain Gene Ontology (GO) Biological Pathway (BP), GO Molecular Function (MF), and Cellular Constituent (CC) sets. In this study, we report BP and MF results, as CC can be less well interpreted with respect to MO. The results with a *P*-value less than 0.005 were considered suggestively significant and genes/pathways with a false discovery rate (FDR) below 0.05 as significant. Correction for whether citalopram was administered in the first or second sampling was included to avoid sampling-day effects; furthermore, age, sex, history of allergy, and smoking status were considered as covariates, where statistically permissible. The following comparisons were made.

##### 2.4.2.1. Comparison of citalopram neuroendocrine challenge effect between patients with migraine without aura and healthy controls

As described in the cortisol analysis, first we calculated the difference of postchallenge minus prechallenge transcriptomic data for both citalopram (DiffCitalopram) and placebo challenge (DiffPlacebo) for each participant. Next, the difference between the citalopram challenge and the placebo challenge was calculated (DiffOfDiffs = DiffCitalopram − DiffPlacebo), again for each participant. Finally, the values of patients with migraine were compared with healthy controls (group comparison). This analysis determined the distinctive effect of citalopram neuroendocrine challenge in patients with MO. To explore sex-specific effects, the procedure was repeated separately for female and male participants.

##### 2.4.2.2. Comparison of citalopram neuroendocrine challenge effect with placebo in patients with migraine without aura

In this analysis, postchallenge reactions of patients with MO to citalopram neuroendocrine challenge were compared with the effect of placebo (the main effect of DiffOfDiffs in patients with MO). Investigating the postchallenge phase in this subgroup helps to pinpoint the effect of citalopram neuroendocrine challenge particularly in patients with MO.

##### 2.4.2.3. Comparison of citalopram neuroendocrine challenge effect with placebo in healthy controls

In this analysis, postchallenge reactions of healthy controls to citalopram neuroendocrine challenge were compared with the effect of placebo (the main effect of DiffOfDiffs in healthy controls). This subgroup analysis helps to identify the effects of citalopram in healthy controls.

Placebo analyses were also conducted prechallenge and postchallenge to address potential baseline differences and differences in the physiological response to the control treatment.

## 3. Results

### 3.1. Descriptive statistics of the population

Details about the recruited 51 participants and *P*-values of statistical tests for differences (conducted with χ^2^, and Mann-Whitney *U* and *t* tests) can be seen in Table 1.

**Table 1:** Descriptive statistics of our cohort.

		Con (MO)						Χ	*P*
a	Male	14(5)						1.87	0.1715
	Female	16(16)							
b	Allergy	Counts Con (MO)	Pollen, weed, dust			Drug	Food		
		8 (9)	4 (7)			3 (2)	1 (0)	0.82	0.3653
c	Age	Mean Con (MO)	Median Con (MO)			SD Con (MO)		U	
		26.38 (26.91)	25.75 (25.63)			4 (5.2)		315	1
d	Smoking	Smoker Con (MO)	1	2	3	4	5		
		7 (21)	23 (0)	4 (13)	0 (4)	2 (4)	1 (0)	556.5	8.75E–08
e	MO onset age	Mean onset age (y)	14.33						
		SD (y)	5.45						
f	MIDAS B scores	Score	5.5						
		SD	1.6						
g	Trigger for migraine attack	Stress	Yes	12					
			No	9					

				t-Value	df	*P*	95% CI	Mean MO	Mean Con
h	Psychological assessment	Basis	SDS	2.02	72.05	0.046	0.0663, 8.8923	37.73	33.25
			STAI-I	2.36	82.91	0.026	0.6272, 7.3343	41.57	37.59
			STAI-S	3.38	77.01	0.001	1.3286, 5.1276	37.09	33.86
		Day 1 and day 2 adjusted from basis	SDS	0.06	91.94	0.95	−1.2670, 1.3495	−2.01	−2.05
			STAI-I	−0.3	93.88	0.76	−2.3385, 1.7200	−2.46	−2.15
			STAI-S	−0.5	88.25	0.62	−4.8331, 2.9042	−2.47	−1.51

The table presents the following: (a) sex distribution, showing absolute numbers of male and female controls and patients with MO with corresponding χ^2^ test statistics comparing patients with MO with controls; (b) Allergy history in MO and controls with pollen, weed, dust, drug, and food as potential allergens and corresponding χ^2^ test statistics; (c) age differences between patients with MO and controls on the day of their citalopram administration and corresponding Mann-Whitney *U* test statistics; (d) smoking status values and corresponding Mann-Whitney *U* test statistics; (e) MO onset age in years; (f) MIDAS B scores in MO; (g) triggers for migraine attacks, specifically stress, with frequencies of “Yes” and “No” responses; (h) psychological assessments, including SDS, STAI-I, and STAI-S scores, with corresponding Welch *t* test statistics. Assessments were conducted: At basis, comparing patients with MO to controls. Day 1 and day 2 were adjusted from basis, with results subtracted from basis scores for days 1 and 2 before blood sampling.

CI, confidence interval; Con, control; df, degrees of freedom; MO, migraine without aura; numbers regarding smoking: 1—never, 2—occasionally, 3—a few cigarettes/day, 4—half box/day, 5—1 or more than 1 box/day; SD, standard deviation; t-value, test statistic from Welch *t* test; χ, chi-square (χ^2^) distribution.

### 3.2. Comparison of citalopram neuroendocrine challenge effect between migraine without aura patients and healthy controls

#### 3.2.1. Citalopram neuroendocrine challenge elicited elevated stress response in migraine without aura patients

**Figure 2.**
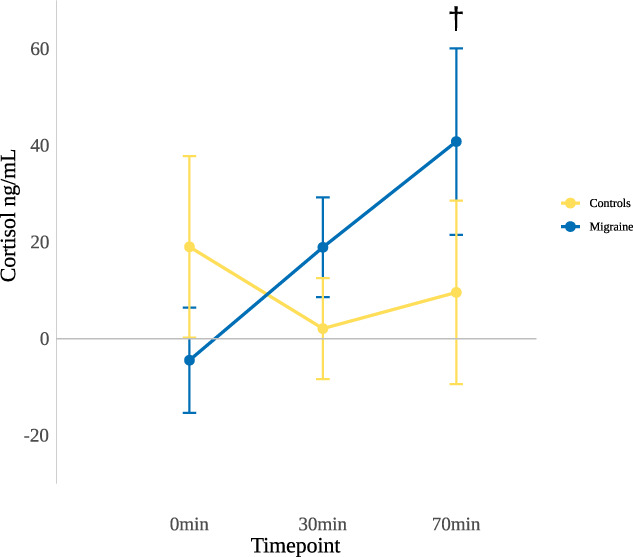
Relative cortisol-concentration changes elicited by citalopram neuroendocrine challenge in healthy controls and patients with MO. Citalopram neuroendocrine challenge effect on plasma cortisol was determined by calculating the difference of the plasma cortisol-concentration postchallenge minus prechallenge for both citalopram and placebo and then subtracting changes after placebo from changes after citalopram. Mann-Whitney *U* test results: 0 minute (*P*-value = 0.13, U = 203); 30 minutes (*P*-value = 0.1355, U = 347); a trend-level significant difference at 70 minutes (*P*-value = 0.07292, U = 361). Whiskers show standard error. Variance in cortisol levels in the migraine group was higher compared with controls, reflecting the inherent heterogeneity in stress sensitivity among patients with migraine. Please note that in patients with MO, the negative mean difference (mean = −4.40 ng/mL, SD = 47.41 ng/mL) at baseline (t = 0 minute) indicates an elevated cortisol concentration before the placebo challenge. †Trend-level significant *P*-value < 0.1. MO, migraine without aura.

To test whether the citalopram challenge was capable of eliciting a larger stress response in patients with MO than in healthy controls, we compared cortisol concentrations before vs after the challenge for individuals taking either placebo or citalopram and tested for a difference between patients with MO and controls both 30 and 70 minutes after the infusion started. The results showed continuously elevating cortisol concentrations in patients with MO, with the difference reaching trend-level significance at 70 minutes (Fig. 2).

The results from placebo responses and sex-specific analysis confirmed that baseline, placebo-elicited, and sex-specific responses bear no or little impact on this result (Supplementary Table 1, https://doi.org/10.5281/zenodo.14910825, Supplementary Table 2, Supplementary Figures 2-4, http://links.lww.com/PR9/A289).

### 3.3. Gene-level results of citalopram neuroendocrine challenge in patients with migraine without aura compared with healthy controls

To determine the underlying molecular correlates of the larger cortisol response, we compared healthy controls and patients after correction for postchallenge minus prechallenge and citalopram minus placebo challenge. The comparison did not yield significant genes after multiple hypothesis corrections. With a suggestive significance below 0.005, however, 10 genes were significant, 2 downregulated, and 8 upregulated (Table 2). Among these, *IKBKGP1* showed the largest downregulation (logarithm fold change [log2FC]_IKBKGP1_ = −6.135, p_IKBKGP1_ = 2.66 × 10^−05^).

**Table 2:** Differential gene expression in response to citalopram neuroendocrine challenge in patients with migraine without aura compared with healthy controls.

HGNC symbol	Description	log2FC	*P*
*IKBKGP1 (IKBKG)*	Inhibitor of nuclear factor kappa-B kinase subunit gamma pseudogene 1	−6.13 (−0.18)	2.669 × 10^−5^ (0.4704)
***ENOX2***	Ecto-NOX disulfide-thiol exchanger 2	0.51	0.0013
*GJA3*	Gap junction protein alpha 3	2	0.0015
—	Novel transcript, antisense to KIAA1012	−1.21	0.0032
*PRKCE*	Protein kinase C epsilon	0.67	0.0032
*RRP15*	Ribosomal RNA processing 15 homolog	0.61	0.0034
*NKRF*	NFκB repressing factor	0.54	0.0042
*ATAD5*	ATPase family AAA domain containing 5	0.88	0.0046
—	Novel transcript, sense intronic to VAMP3	1.31	0.0047
*TTC39A*	Tetratricopeptide repeat domain 39A	1.48	0.0048

Ten differentially expressed genes with suggestive significance (*P*-value < 0.005) in patients with MO compared with healthy controls were determined by calculating the postchallenge minus prechallenge values in the placebo challenge subtracted from the postchallenge minus prechallenge values in citalopram challenge and comparing the 2 groups. For pseudogenes, logFC and significance values of parent genes and pseudogenes combined together can be found in parentheses.

HGNC symbol, HUGO human gene nomenclature committee symbol; log2FC, log2 fold change.

Analysis of the MO and control groups separately showed that the top gene in the main comparison, *IKBKGP1*, has been downregulated in patients with MO. By contrast, in the control group, genes showed marginal changes (Supplementary Table 1, https://doi.org/10.5281/zenodo.14910825) after the citalopram challenge, with some exceptions, like, eg, *NKRF*, being downregulated in healthy controls and upregulated in patients (Supplementary Table 1, https://doi.org/10.5281/zenodo.14910825). Additional genes were also suggestively significant with opposite direction of changes in MO and healthy control groups usually below or close to nominal significance level, representing divergent alterations in the 2 groups.

### 3.4. Pathway-level results of citalopram neuroendocrine challenge in patients with migraine without aura compared with healthy controls

Comparison of citalopram and placebo postchallenge vs prechallenge in patients with MO with postchallenge vs prechallenge in controls with correction for sampling-day, age, sex, smoking, and allergy status yielded no FDR-significant C2 and C5 pathways and 472 FDR-significant positively enriched C3 pathways. Altogether 7, 420, and 10 suggestively significant C2, C3, and C5 gene sets were found (Table 3, Supplementary Table 1, https://doi.org/10.5281/zenodo.14910825). Inspection of C5 sets indicated an upregulation in protein synthesis and carbohydrate metabolic processes and negative enrichment of phototransduction. Sex-specific analyses showed a “filtering” effect of male results, potentially, due to lower statistical power.

**Table 3:** Enriched gene pathways in response to citalopram neuroendocrine challenge in patients with migraine without aura compared with healthy controls.

Pathway	*P*	log2err	NES
Ribonucleoprotein complex biogenesis	0.0006	0.4773	1.4526
Positive regulation of carbohydrate metabolic process	0.0007	0.4773	1.6680
Positive regulation of B cell proliferation	0.0008	0.4773	1.6905
Cytoplasmic translation	0.0015	0.4551	1.5760
Phototransduction	0.0015	0.4551	−1.7453
ATP hydrolysis activity	0.0021	0.4317	1.4680
Regulation of B cell proliferation	0.0023	0.4317	1.6223
tRNA modification	0.0033	0.4317	1.5724
Positive regulation of cellular carbohydrate metabolic process	0.0034	0.4317	1.6328
Alpha–amino acid metabolic process	0.0036	0.4317	1.5387

Findings of the enrichment analysis of gene pathways, after comparing postchallenge vs prechallenge effects of citalopram in patients with MO and controls. The C5 examination of pathways highlighted overexpression in pathways of protein synthesis, phototransduction, and processes related to amino acids and carbohydrates. Important metrics of enrichment are presented.

log2err, log fold error rate; MO, migraine without aura; NES, normalized enrichment score.

### 3.5. Comparison of citalopram neuroendocrine challenge effect with placebo separately in patients with migraine without aura and in healthy controls

Subgroup analysis of citalopram neuroendocrine challenge in comparison to placebo in patients with MO revealed 21, 544, and 11 gene sets from the C2, C3, and C5 sets, respectively, at suggestive significance level and 9, 656, and 1 FDR-significant gene sets (Table 4 and Supplementary Table 1, https://doi.org/10.5281/zenodo.14910825). C2 and C5 sets confirmed an upregulation in translation in patients with MO without comparisons with controls. Investigating the citalopram challenge in healthy controls, almost all of these processes were slightly, nonsignificantly downregulated. There were no FDR-significant gene sets found in healthy controls. The results confirmed that most differences in the comparison between patients with MO and controls reflect dysregulation of processes in MO. Full results are in Supplementary Table 1, https://doi.org/10.5281/zenodo.14910825.

**Table 4:** Enriched gene pathways in response to citalopram neuroendocrine challenge in patients with migraine without aura.

Pathway	*P*	log2err	NES
Structural constituent of ribosome	2.564 × 10^−6^	0.6272	1.7790
Cytoplasmic translation	3.763 × 10^−5^	0.5573	1.7172
Ribonucleoprotein complex biogenesis	4.450 × 10^−5^	0.5573	1.5580
Ribonucleoprotein complex subunit organization	0.0001	0.5188	1.6121
tRNA modification	0.0008	0.4778	1.6657
Response to lectin	0.0011	0.4550	1.7049
Polypyrimidine tract binding	0.0012	0.4550	1.6543
mRNA binding	0.0020	0.4317	1.4786
Positive regulation of natural killer cell-mediated cytotoxicity	0.0041	0.4070	1.6090
ncRNA processing	0.0046	0.4070	1.3650
Catalytic activity acting on RNA	0.0046	0.4070	1.3543

Pathway enrichment subgroup analysis outcomes in patients with MO after citalopram neuroendocrine challenge at suggestive significance level. This table includes results for C5 (Gene Ontology pathways) gene sets. The C5 sets involve enrichment in translation, and consequently, they support the proposed upregulated activity of these processes in migraine.

log2err, log fold error rate; MO, migraine without aura; NES, normalized enrichment score.

### 3.6. Effect of inclusion of patient with migraine attack

The gene-level analysis identified 9 suggestive significant genes, 6 overlapped with results excluding the patient. Pathway-level analysis revealed 3 pathways in the C2 and C5 sets and 355 in the C3 set showing overlaps, between the 2 analyses. Pathways, such as glycosylation and glycoprotein processes, achieved suggestive significance when the attack data were included and were nominally significant in the data set excluding the patient, with overall patterns and directions of change consistent. For full results, see cortisol level analyses, including Supplementary Figure 5, http://links.lww.com/PR9/A289.

## 4. Discussion

In this study, we demonstrated a difference between patients with MO and controls in their cortisol response to an acute neuroendocrine challenge induced by citalopram infusion. Underlying molecular correlates associated with the elevated stress response pointed to a reduction in *NF*κ*B* regulation, initiation of cellular protein synthesis, and an upregulation in carbohydrate metabolism-related pathways. These results may point to possible early molecular mechanisms that lead to a migraine attack after stressors in MO and demonstrate an altered stress reaction in patients.

Elevation of cortisol following acute stress is considered as a modulator to reinstate homeostasis by providing energy resources, restricting potentially dangerous immunological responses,^42^ and re-establishing a normal affective state.^20^ Accordingly, after stressors, salivary and blood cortisol concentrations are often used as biomarkers for stress response.^22,35^ For citalopram neuroendocrine challenge, temporal patterns of cortisol differ slightly from psychosocial stressors,^11^ due to its specific neuroendocrine characteristics. In healthy humans, i.v. 10 mg citalopram administration gradually led to a continuous and slight elevation until 150 minutes, with a small decrease at 30 minutes in blood cortisol concentrations, when compared with placebo.^31^ Very similar changes were observed in this study in healthy controls after 7.5 mg citalopram infusion (Fig. 1). However, patients with MO showed a different cortisol response, namely, cortisol concentrations in patients elevated continuously and reached trend-level significance at 70 minutes, indicating an increased stress reactivity in MO and the potential abnormal functioning of the HPA axis.

Our results, together with an elevated STAI and SDS scores in patients, further support the previous hypothesis that a maladaptive stress response is an important factor in the recurrence of migraine attacks^6^ and are in line with the observation that altered HPA activity, especially in the anterior hypothalamus that orchestrates responses for environmental stimuli with endocrine and metabolic changes, precedes migraine attacks.^44,45^

Cortisol mainly exerts its effects through glucocorticoid (GR) and mineralocorticoid receptors. The low-affinity GRs, after dissociating from HSP90 by binding cortisol, form dimers and after arriving in the cell nucleus influence gene expression of genes with responsive elements.^10^ In healthy individuals low-dose hydrocortison administration caused the dysregulation of approximately 100 genes already at 1 hour after administration in peripheral blood mononuclear cells. Larger doses caused larger gene expression alterations.^38^

In this study, in patients with MO, 10 genes were differentially expressed after the neuroendocrine challenge, among them the pseudogene, *IKBKGP1*. *IKBKGP1* is a nonprocessed, nontranscribed pseudogene.^1,3,14,32^ While we cannot exclude completely that in certain situations (like migraine) pseudogenes or parts of pseudogenes are processed, to mitigate ambiguities, we provided corrected log2FC value, which was calculated by combining pseudogene counts and parent gene counts into 1 “hybrid” gene and performing subsequent analysis steps with the hybrid gene to obtain log2FC and significance values (Table 2). The calculated values showed that *IKBKGP1* becomes nonsignificant using the combined gene, albeit, its direction of change remains consistent.

Among the dysregulated genes, both *IKBKG* and *NKRF* are associated with *NF*κ*B*. The parent gene of *IKBKGP1*, *IKBKG* (*NF*κ*B* essential modulator [NEMO]/inhibitor of nuclear factor kappa-B kinase subunit gamma) is a regulatory element capable of inducing the canonical *NF*κ*B* activation pathway.^23^ *NKRF* is a transcriptional silencer known to repress transcription of several *NF*κ*B*-responsive genes.^13^ In our study, we have found a downregulation of *IKBKGP1* and an upregulation of *NKRF* suggesting a diminished *NF*κ*B* activation and transcriptional repression of *NF*κ*B* targets, respectively, in MO. There is a mutual antagonistic regulation between *NF*κ*B*, its downstream targets, and cortisol.^4^ For example, hydrocortisone-induced gene expression changes conferred downregulations in *NF*κ*B* and apoptosis-related pathway-associated genes, including numerous interleukins.^38^ Thus, the neuroendocrine-challenge-induced, elevated cortisol concentration may exert its negative effect on *NF*κ*B* and its targets at this early time point in patients with MO through the highlighted, dysregulated gene(s), especially *NKRF*.

However, in pathway-level analyses, *NF*κ*B*-related pathways remained insignificant. The pathways directly related to immune processes were the upregulated “Positive regulation of B cell proliferation” and “Regulation of B cell proliferation” in patients with MO compared with healthy individuals. This was probably catalyzed by the suggestively significant dysregulation of *ATAD5*, a gene known to regulate B cells.^56^ Irrespective of the underlying cause, on a larger scale, the negative regulation of *NF*κ*B* and related immune processes expected from a larger cortisol response failed to materialize. This discrepancy may be explained by the early time point used in this study when cortisol could not yet exert its full-scale effects.

Despite this early timeframe after the citalopram neuroendocrine stress challenge, some changes indicated the start of a cellular program in patients with MO. Mitochondrial- and energy-delivering gene *ENOX2* was upregulated. The parallel upregulation in protein synthesis-related pathways, including cytoplasmic translation and ribonucleoprotein complex biogenesis suggest a starting cellular protein synthesis at this time point in blood. Given that cortisol is a well-known and influential transcriptional regulator, the above changes may reflect the initiation of transcriptional/translational processes by the larger, evoked cortisol response in patients with MO. This is in line with previous evidence indicating an altered amino acid profile associated with migraine status.^19^ In support results in patients with migraine showed, indeed, translation- and amino acid–related gene sets from both C2 and C5, some reaching FDR significance (Supplementary Table 1, https://doi.org/10.5281/zenodo.14910825).

In addition to C5 sets, C3 and C2 gene set analyses delivered limited further insights. The results from C2 gene sets were either supportive of C5 results, or hard to interpret in light of MO. The C3 set analyses showed alterations that further confirmed cortisol actions. For example, abundant upregulations among genes regulated by microRNAs that are under cortisol control were observed.^9^ These alterations included significant upregulation of miR-511-3p-, miR-98-5 p-, and additional let7 family-regulated genes (Supplementary Table 1, https://doi.org/10.5281/zenodo.14910825) and may reflect extensive recruitment of miRNAs in the changes following cortisol-concentration elevations in patients. We have to note that among migraine-specific miRNAs,^15,16^ miR-181a was also identified in our study among significant C3 sets, indicating that cortisol actions may induce some migraine-specific changes.

Our study, accidently, provided a unique opportunity to measure citalopram-elicited changes after a migraine attack. Many of the above alterations were consistent, eg, translation-related processes and *IKBKGP1/NKRF* dysregulations; yet, an important difference emerged in the marked and consistent upregulation in pathways related to glycosylation and glycoprotein biosynthetic processes, in parallel with larger differences in cortisol values between controls and patients. The observed alterations may reflect the protective role of glucocorticoids in the maintenance of the endothelial glycocalyx,^7,8^ an important constituent of all endothelial walls, composed of glycoproteins, proteoglycans, and soluble components.^41^ The glycocalyx prevents access of circulating inflammatory cells to the endothelium and its receptors and, thereby, inhibits inflammation.^43^ Glucocorticoids are able to prevent degradation of the glycocalyx. Hydrocortisone, eg, (1) prevented shedding of major glycocalyx components^7^ and (2) prevented TNF-alpha-induced inflammatory degradation.^8^ The found upregulations in glycoprotein and carbohydrate metabolism with the patient with an attack, thus, may represent a consequence of the elevated cortisol concentrations suppressing immunological vascular reactivity. However, the glycocalyx is an important contributor to NO-induced vasodilation.^43^ Studies demonstrated that diminished glycocalyx components are associated with reduced or absent NO-induced vasodilation.^28,33,55^ Given that NO is known to induce human migraine attacks, increased glycoprotein and proteoglycan production may provide a link connecting elevated cortisol concentrations with the attack.^36,37,40^ Indeed, following the neuroendocrine challenge, the patient's status gradually deteriorated to the point, when i.v. medications were administered. Taken together, increased glycoprotein and proteoglycan production may provide a link, to how the elevated cortisol response can evoke/worsen migraine attacks and provide an interesting direction for future research.

Our study comes with limitations. (1) Future studies should address the temporal trajectory of the RNA-seq alterations and long-term cortisol responses to obtain a fuller picture of the identified genes and pathways. (2) Citalopram challenge does not completely correspond to psychosocial stress, as citalopram acts through the serotonergic system as described in Reference 18. Future studies need to dissect such effects. (3) Patients with aura, who may demonstrate differences from the findings presented here, should be evaluated in future attempts. (4) Additional populations may also be tested to generalize findings using other large and well-characterized samples. (5) Additional immunological molecules may be implicated in glucocorticoid-induced altered glycosylation patterns,^54^ therefore, our interpretation of the role of glycocalyx is not comprehensive. (6) Sex-specific analyses showed a larger variation in cortisol levels in male patients with migraine and in female controls, and more suggestively significant genes in women, reflecting potential underlying hormonal/sex influences.^27^

In conclusion, this study demonstrated elevated cortisol concentrations in patients with MO in comparison to healthy controls in response to an acute citalopram neuroendocrine challenge. Parallel molecular patterns on the transcriptomic level reflected changes well explained by cortisol's known effects. Among these, an initiation of a cellular protein synthesis program and the increased carbohydrate metabolism may reflect a starting altered production of glycocalyx constituents, eventually increasing NO-induced vasodilation and becoming manifest during stress-related MO attacks.

## Disclosures

The authors have no conflict of interest to declare.

## Supplemental digital content

Supplemental digital content associated with this article can be found online at http://links.lww.com/PR9/A289, https://doi.org/10.5281/zenodo.14910825.

## Supplementary Material

SUPPLEMENTARY MATERIAL
