# Supplementary material for: Acute neuroendocrine challenge elicits enhanced cortisol response and parallel transcriptomic changes in patients with migraine
Source: Pain Rep. 2025 May 1;10(3):e1254. doi: 10.1097/PR9.0000000000001254 (PMC12047896; doi:10.1097/PR9.0000000000001254)
Supplement: SUPPLEMENTARY MATERIAL [file painreports-10-e1254-s001.pdf]

Supplementary figure 1. Cortisol concentration differences

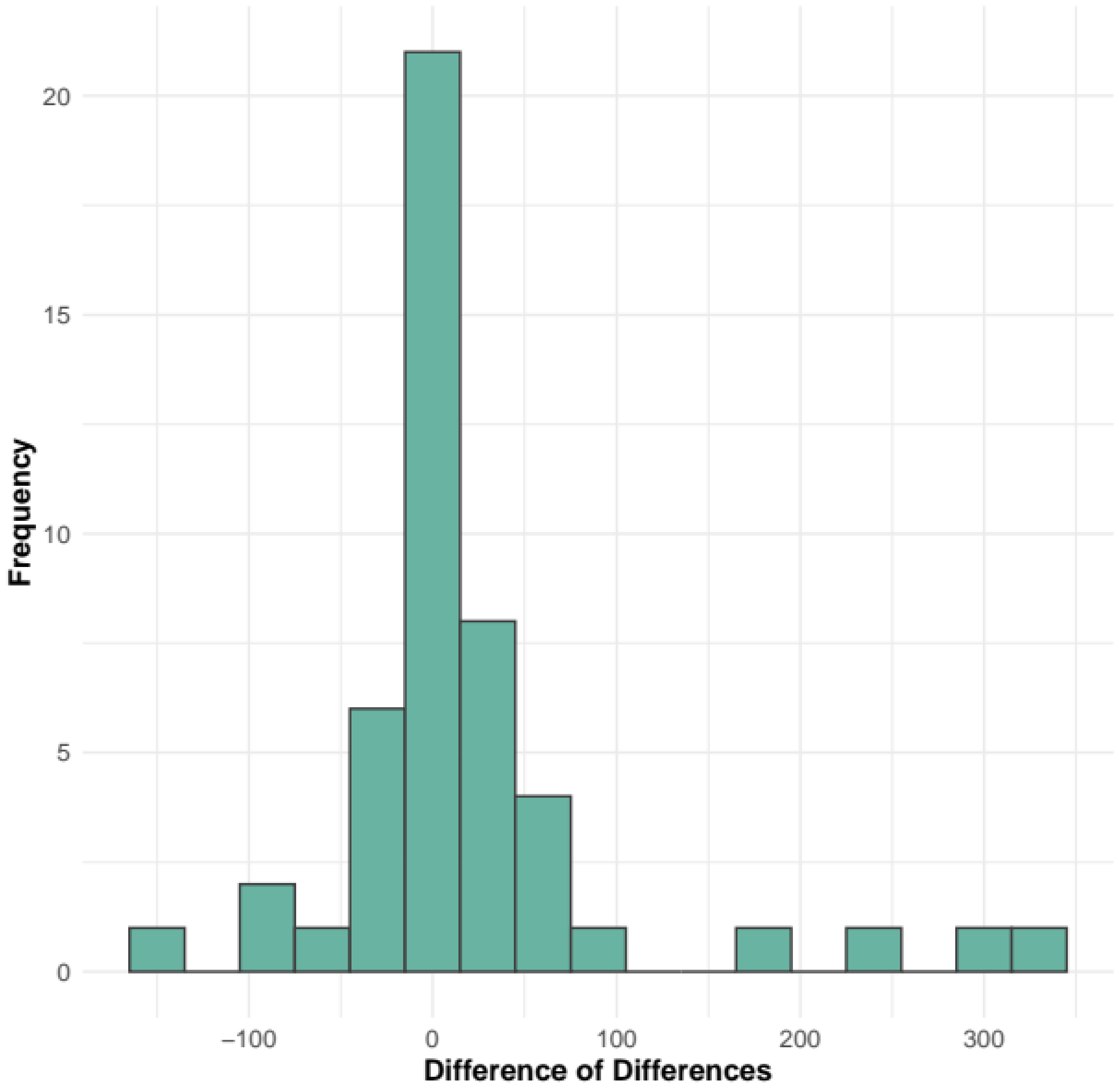

Comparison of cortisol concentration differences between pre- and post-challenge measurements for citalopram (DiffCitalopram) and placebo (DiffPlacebo) in migraine without aura (MO) patients and healthy controls. The figure shows the calculated differences of the differences (DiffOfDiffs = DiffCitalopram - DiffPlacebo) between the two groups. Statistical significance was determined using the non-parametric Mann-Whitney-U test due to non-normal data distribution. Data represent mean values with standard deviation.

Supplementary figure 2. Neuroendocrine challenge cortisol measures in females

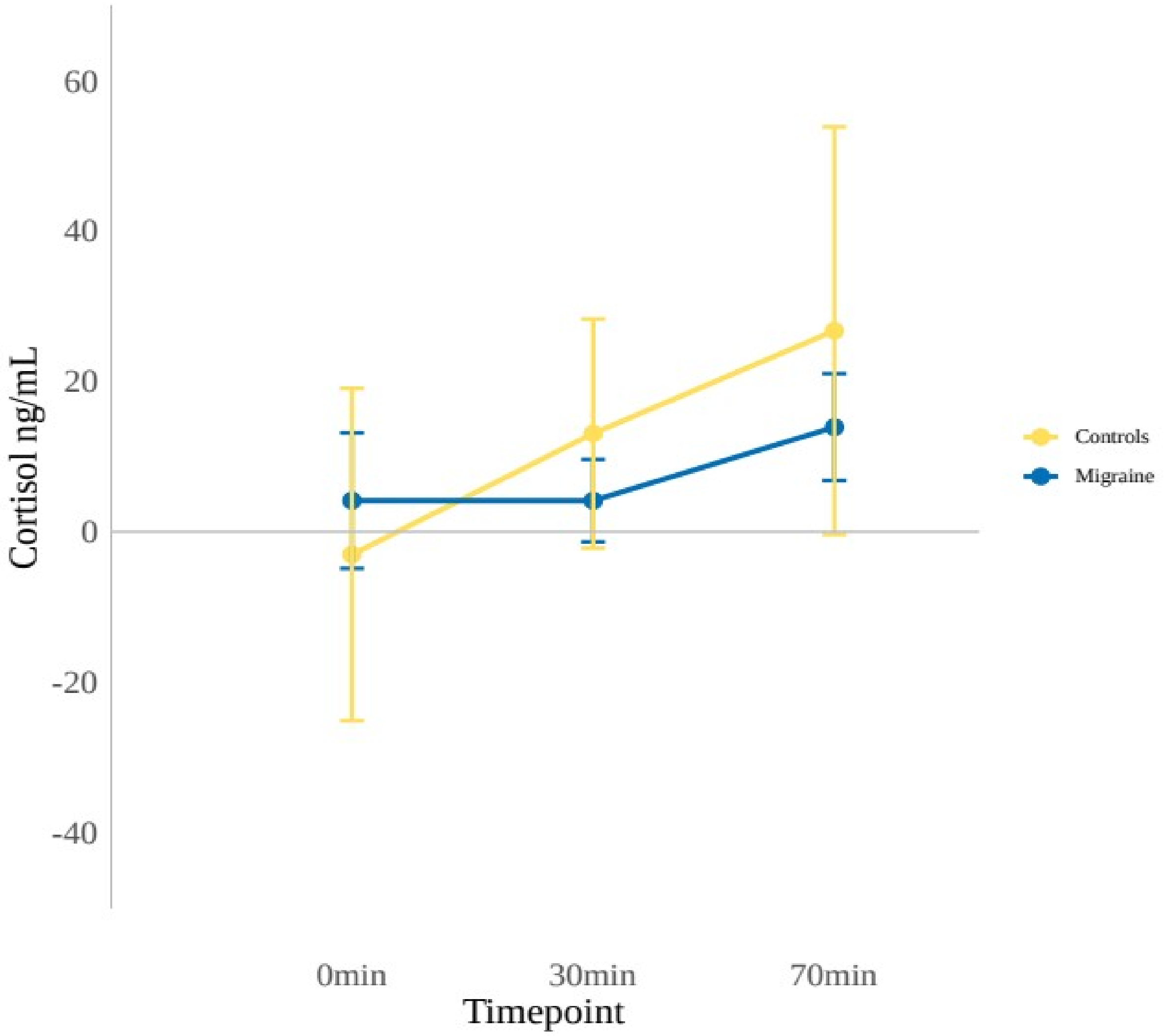

Effects of citalopram neuroendocrine challenge in females on plasma cortisol was determined by calculating the difference of the plasma cortisol concentration post-challenge minus pre-challenge for both citalopram and placebo, and then subtracting changes after placebo from changes after citalopram. Comparison between female MO patients and healthy controls show no siginificant results between both groups on all time points. Whiskers show standard error. MO – Migraine without aura

Supplementary figure 3. Neuroendocrine challenge cortisol measures in males

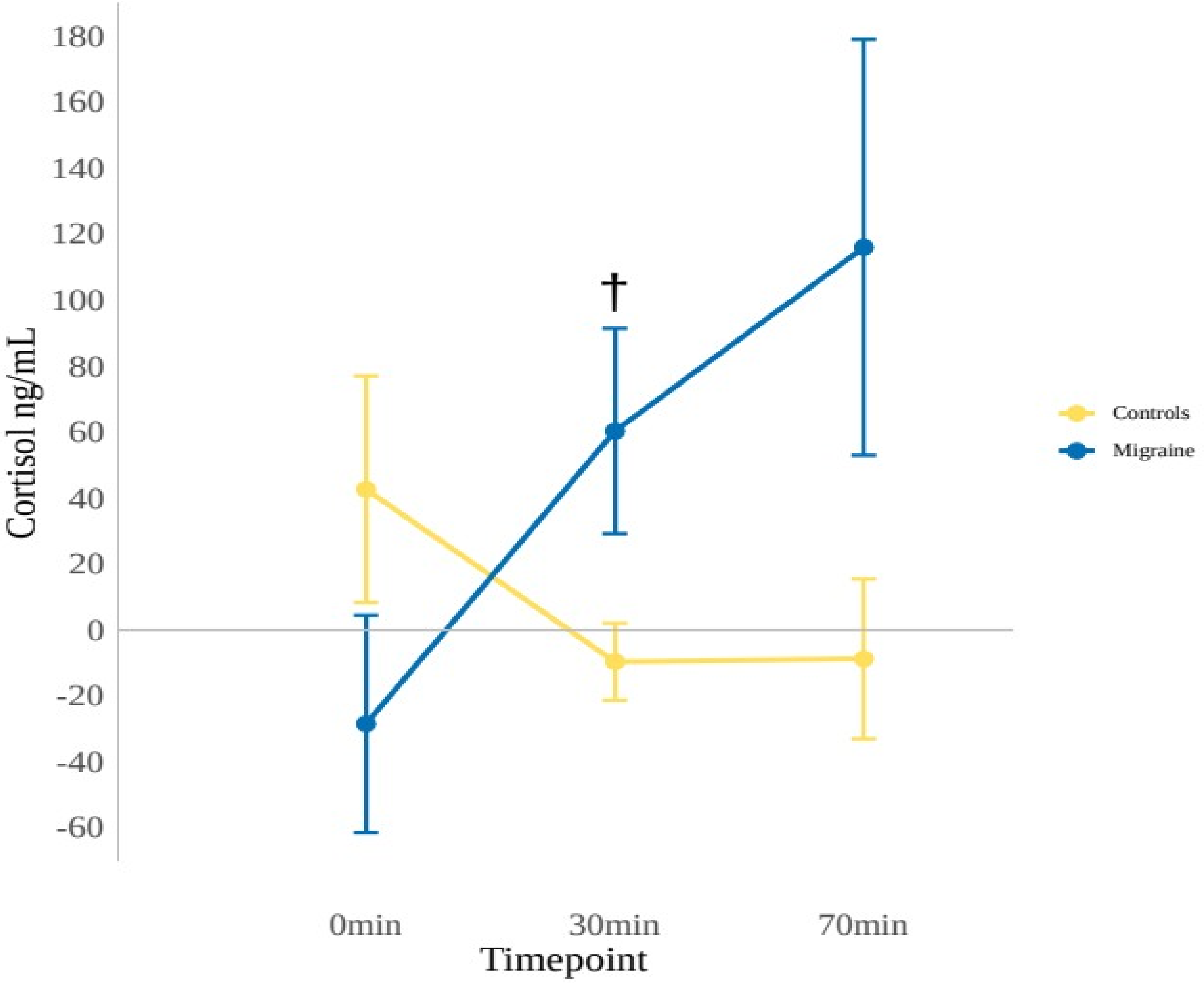

Effects of citalopram neuroendocrine challenge in males on plasma cortisol was determined by calculating the difference of the plasma cortisol concentration post-challenge minus pre-challenge for both citalopram and placebo, and then subtracting changes after placebo from changes after citalopram. Significant difference between MO patients and healthy controls was determined by non-parametric Mann-Whitney-U test. Whiskers show standard error. Comparing male MO and healthy controls no siginificance could be detected. MO – Migraine without aura, † – trend-level significant p-value<0.1

Supplementary figure 4. Dotted projection of Figure 2

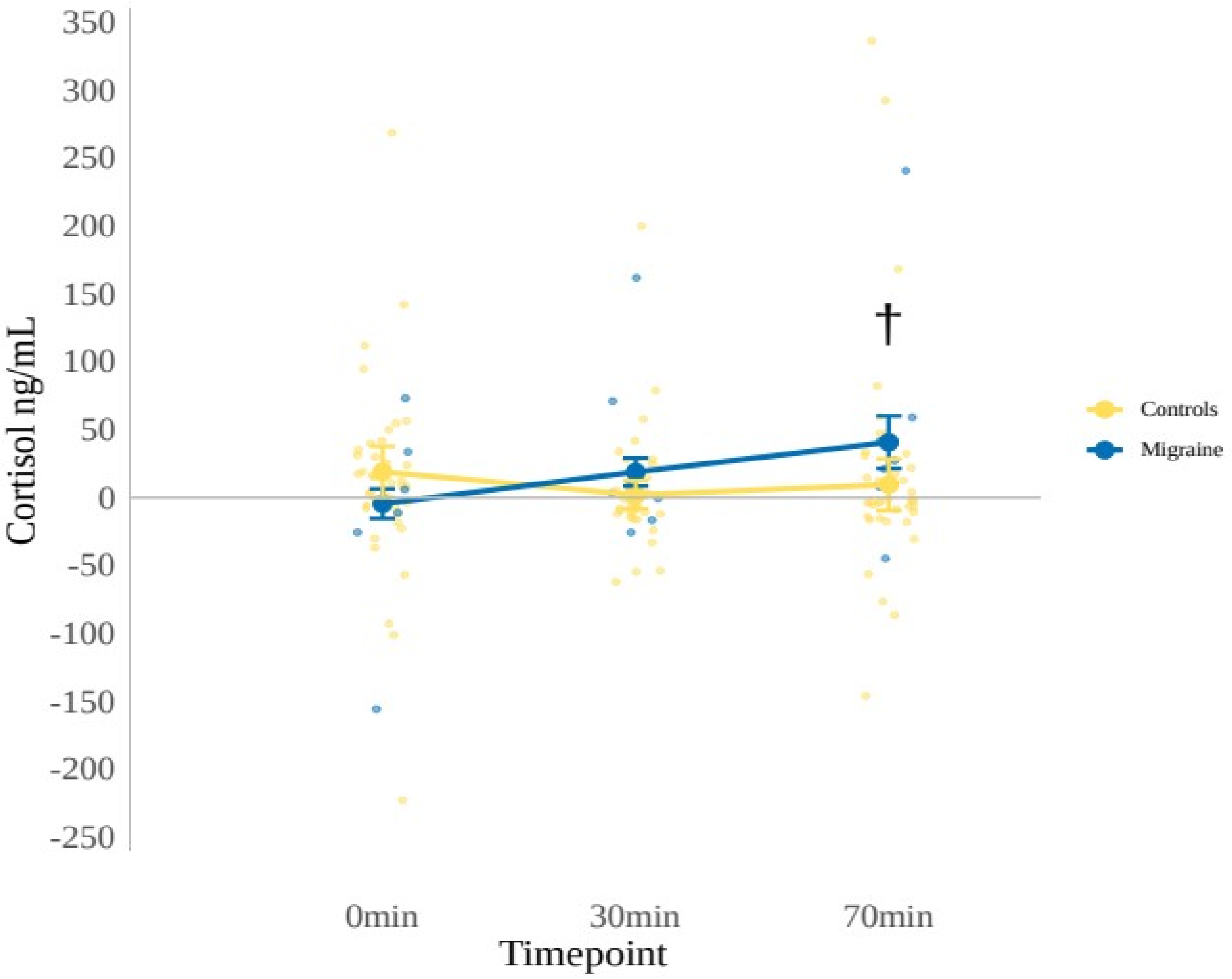

This figure provides an extended visualization of the data from Figure 2, including a dotted projection to illustrate individual data points. The high variance observed originates from multiple subjects, reflecting the heterogeneous sensitivity to stress in the migraine group.

Supplementary figure 5. Cortisol measurements with the inclusion of the patient with a migraine-attack

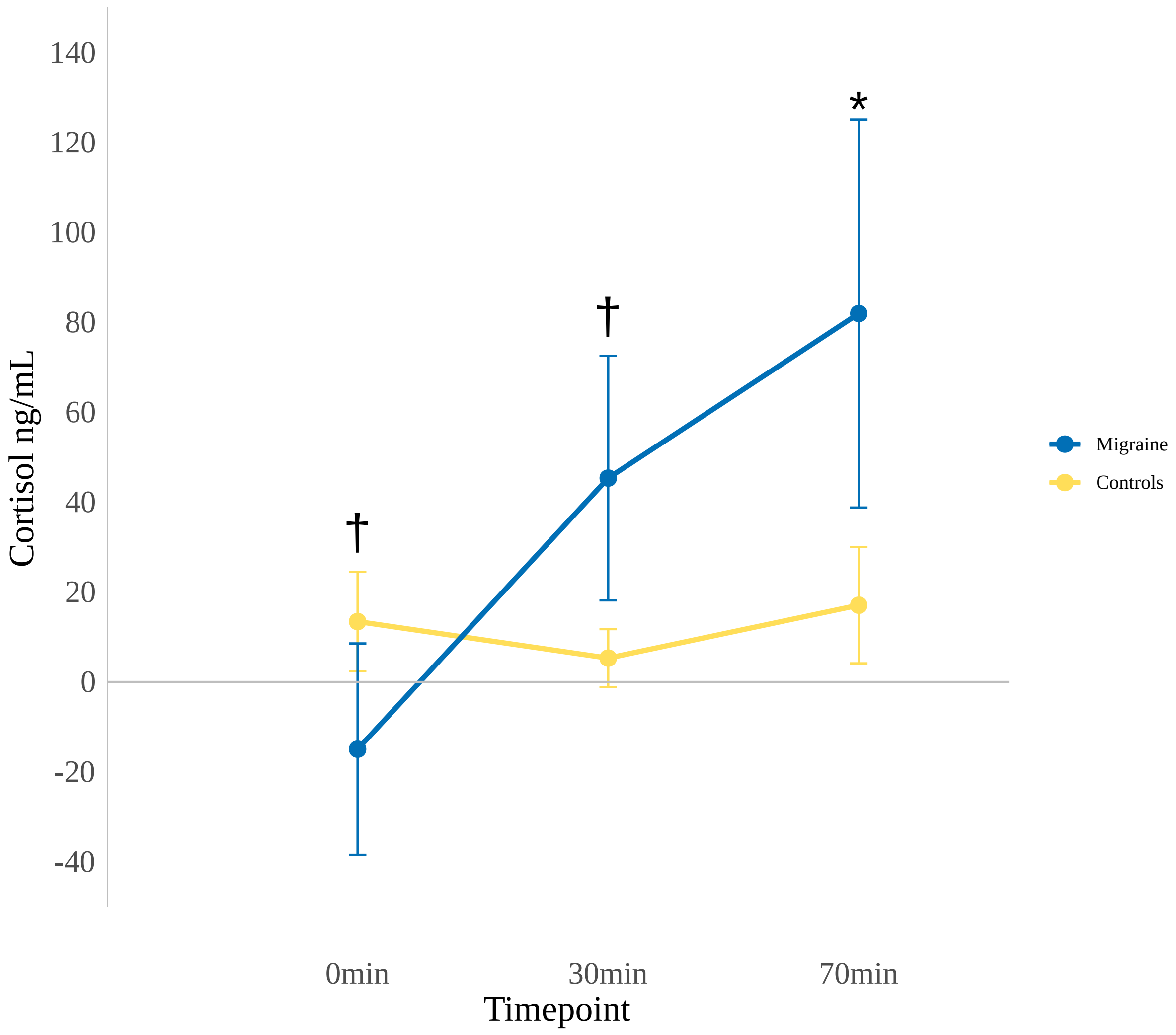

Citalopram neuroendocrine challenge effect, with the inclusion of the attack patient, on plasma cortisol was determined by calculating the difference in the plasma cortisol concentration post-challenge minus pre-challenge for both citalopram and placebo, and then subtracting changes after placebo from changes after citalopram. Trend-level significance was observed at 0 minutes (p-value = 0.08953, U=206) and 30 minutes (p-value = 0.086, U = 375) and a significant difference at 70 minutes (p-value= 0.044, U= 389), which was determined by non-parametric Mann-Whitney-U test. Whiskers show standard error. Please note, that in MO patients the negative mean difference (mean=-5.96 ng/mL, SD=46.66 ng/mL) at baseline (t=0 min) indicates an elevated cortisol concentration before the placebo challenge. * – p-value<0.05, † – trend-level significant p-value<0.1, MO – Migraine without aura

Supplementary table 2 Gender separated cortisol differences

Gender	Time (min)	U	p-value	Standard Deviation MO(Con)
Male	0	17	0.1068	73.60
				76.76
	30	56	0.05573	69.57
				26.08
	60	51	0.156	141.11
				54.25
Female	0	95	0.683	33.81
				82.76
	30	113	0.7472	20.51
				57.02
	60	130	0.2898	26.58
				101.48

The table shows: Gender separated citalopram neuroendocrine challenge effect on plasma cortisol was determined by calculating the difference of the plasma cortisol concentration post-challenge minus pre-challenge for both citalopram and placebo, and then subtracting changes after placebo from changes after citalopram. No significant difference between MO and Con was determined by non-parametric Mann-Whitney-U test. Con – Control; MO – Migraine without aura
